# An Attempt to Predict the Preferential Cellular Orientation in Any Complex Mechanical Environment

**DOI:** 10.3390/bioengineering4010016

**Published:** 2017-02-22

**Authors:** Cédric P Laurent, Jean-François Ganghoffer, Rachid Rahouadj

**Affiliations:** CNRS, LEMTA, UMR 7563, Université de Lorraine, 2 Avenue de la Forêt de Haye, 54502 Vandoeuvre-lès-Nancy, France; jean-francois.ganghoffer@univ-lorraine.fr (J.-F.G.); rachid.rahouadj@univ-lorraine.fr (R.R.)

**Keywords:** mechanobiology, cell mechanics, mechanosensing, scaffold, numerical simulation

## Abstract

Cells respond to their mechanical environment in different ways: while their response in terms of differentiation and proliferation has been widely studied, the question of the direction in which cells align when subject to a complex mechanical loading in a 3D environment is still widely open. In the present paper, we formulate the hypothesis that the cells orientate in the direction of unitary stretch computed from the right Cauchy-Green tensor in a given mechanical environment. The implications of this hypothesis are studied in different simple cases corresponding to either the available in vitro experimental data or physiological conditions, starting from finite element analysis results to computed preferential cellular orientation. The present contribution is a first step to the formulation of a deeper understanding of the orientation of cells within or at the surface of any 3D scaffold subject to any complex load. It is believed that these initial preferential directions have strong implications as far as the anisotropy of biological structures is concerned.

## 1. Introduction

Tissue engineering and cell-based therapies constitute a promising alternative to current therapies in the repair of numerous biological tissues. One first milestone in the challenge of regenerating complex tissues is to understand the behavior of isolated cells, particularly when subject to external mechanical loading. It is well known that stem cells respond to their mechanical environment in different ways: particularly, cell proliferation, differentiation, and migration depend on substrate properties and external loading (for recent examples see [1,2]). The biochemical mechanotransduction principles underlying these reactions have been largely studied over the last decade (for recent reviews, see [3,4,5]). Another outstanding characteristic of adherent cells’ response to external mechanical load is their ability to reorient along a particular angle when the substrate is subject to cyclical stretching. This specificity may play a crucial role in the formation of the most efficient functional configuration during tissue development via the production of an anisotropic collagen network [6].

Indeed, it has been clearly observed that adherent cells align in particular directions when they are seeded on a 2D substrate subject to different uniaxial or biaxial stretching [7,8,9,10,11,12]. For instance, experiments performed on elastic membranes [9,10,11,12,13,14] have emphasized that cells align in particular directions in the range of 60°–70° with respect to the stretching direction when simple elongation was applied to the membrane, while they stay aligned in a random manner in the case of equibiaxial stretching, and they align perpendicular to the stretching direction when pure uniaxial stretching is applied (i.e., locked transverse compaction) [11]. Intuitively, this reorientation has been observed and interpreted as a “stretch avoidance reaction” [7,15]. The idea that cells align in the direction of zero strain has been corroborated by further experiments [14,16] performed on stretched 2D elastic substrates.

Simple models have been proposed in order to predict the direction along which adherent cells align when subject to 2D stretching [10,14]. More recently, a new model based on the relaxation of the elastic energy density stored in the cell has been proposed [8], resulting in a good agreement with the experimental data concerning cell reorientation on a 2D substrate subject to biaxial stretching. Nevertheless, the proposed theories are only applicable if one wants to predict the cellular preferential orientation on a linear elastic 2D substrate subject to simple well-known mechanical loading. Moreover, the prediction ability of these models depends on the fitting of parameters (corresponding for example to cell anisotropy [8] or strain thresholds [10,14]), which would be hard to determine in a more general case. Consequently, the question of the direction in which cells align when subject to any complex mechanical environment and when seeded on any 3D scaffold with a potentially complex constitutive law is still widely open.

It has been observed that the preferential cellular orientation was identical whether the substrate is contracted or stretched [10]. The authors concluded that “the deformation signal responsible for cell reorientation was the peak-to-peak axial strain”. In another study [17], it was proposed that vascular cells orientate in such a way that they are subject to the smallest “range of cells shortening or lengthening” for a maximal vessel contraction. Expressing these ideas from a mechanical point of view, it may be therefore stated that cells align along the direction of unitary stretch, which may be easily computed from the right Cauchy-Green tensor. 

It is our assumption in the present paper that cell preferential orientation is guided by the direction of unitary stretch for a given 3D mechanical state (when such a direction exists). In the next section, we firstly present how the direction of unitary stretch in a given mechanical environment may be computed from the results of finite element (FE) analysis. Then, we corroborate our hypothesis by computing the preferential cellular orientation in different simple cases corresponding to either available in vitro experimental data or physiological conditions. As an illustration of the proposed method to determine cellular orientation in any complex mechanical environment, we further compute cell orientations at the surface of a trabecular bone sample subject to a compression loading. We conclude by discussing the consequences of our contribution, as well as its limitations and perspectives. 

## 2. Materials and Methods 

### 2.1. Calculation of Unitary Stretch and Numerical Implementation

In a continuum media subject to a given displacement field u, the right Cauchy-Green tensor can be easily determined from the relation (for more details, refer to any reference book of continuum mechanics such as [18] or [19]):

(1) $$C=F^{T}\cdot F\;\mathrm{with}\;F=I+Grad\;u$$

This lagrangian symmetric tensor corresponds to the transformation of a dot product between two material vectors from a reference configuration to a deformed configuration. It enables the calculation of the stretch λ in any direction starting from a unit vector A in the reference configuration according to the relation:

(2) $$\lambda^{2}\left(A\right)=A\cdot C\cdot A$$

We note that a unitary stretch corresponds to neither compression nor tension in the direction A. Consequently, from the computation of the Cauchy-Green tensor field, the directions in which the stretch is unitary (i.e., no lengthening or shortening) can be determined by minimizing the quantity |λ(A)−1| for a wide range of directions A. 

### 2.2. Numerical Implementation

In the following examples, the tensor field C was determined from the computation of the Green-Lagrange strains by using the FE code FEBio (see for example [20]). The pre- and post-treatment were realized using dedicated in-house algorithms. For each mesh node where C was determined, the stretch was calculated from Equation (2) in different directions of space located in a plane tangent to the surface of the meshed object. The directions A for which the amount |λ(A)−1| were null were then computed and represented. As previous studies suggest, there may be a strain threshold (evaluated at 3.5% [10]) under which the cells do not reorient. Consequently, the preferential direction was set to a random angle when no direction respecting the condition |λ(A)−1|≤0.035 was found.

### 2.3. Planar Case: Stretching of a Membrane

The evolution of cellular orientation when seeded on a 2D substrate subject to simple cyclic stretching states has been largely assessed. Particularly, it has been observed that cells reorient along “two mirror-image angles” with respect to the stretching direction in the case of simple extension (i.e., transverse compaction is free). Depending on the studies [8,9,10,11,12,13,14], angles in the range of about 60°–70° with respect to the stretching direction have been observed. In order to apply our assumption and implementation to this simple case, and based on the work of [11], we considered an elastic membrane subject to (1) simple extension; (2) pure uniaxial stretch; (3) equibiaxial stretch. Cellular preferential orientation of the cells seeded on this 2D substrate were computed according to the above procedure. A 10% strain was applied for these three cases, as proposed in [8,12]. The membrane was considered as a Neo-Hookean material: the FE code FEBio thus required two material parameters, and values of 0.38 [10] and 1 MPa [8] have been chosen respectively for Poisson’s ratio and Young’s modulus of the elastic membrane. The membrane size was 10 × 30 mm^2^ with a thickness of 0.25 mm [12]. The distribution of orientation angles with respect to the stretching direction was plotted and compared to the reported values.

### 2.4. Physiological Case: Inflation of an Arterial Segment

In order to assess the implication of our methodology in the case of non-planar loadings, an inflation test was modelled on an arterial segment. A 2 mm-long arterial segment was modelled as two layers standing for the media and the adventitia. The blood pressure has been estimated to 13 kPa (100 mm Hg, assumed to be the mean arterial pressure as proposed in similar studies [21,22]). The dimensions of the two layers were taken from [23]. Layers were considered to be isotropic linear elastic and nearly incompressible; the elastic modulus of the media was taken from [24], and the ratio between adventitia and media elastic properties was taken from [23]. The preferential cell directions were computed at the inner surface of the media and the outer surface of the adventitia following the above procedure. The orientation angle was expressed with respect to the transverse plane of the arterial segment to be consistent with the angle used in the literature (see for instance [25]). 

### 2.5. Illustration for a Complex 2D Mechanical Environment: Stretching of a Perforated Membrane

As an illustration of the above procedure to more complex 2D mechanical environments, the cellular preferential orientation was computed at the surface of a perforated elastic membrane subject to a 10% uniaxial stretch. This simple case has the particularity to lead to heterogeneous strains (both tensile and compressive) and to be experimentally testable. It may also correspond to the case of wound healing, which has been the subject of numerous studies recently due to its implication in scar formation [26,27]. The silicone membrane was modelled as a Neo-Hookean material as described above, and had the same geometry as the above membrane, except that a 5 mm hole was added in the centre of the membrane. 

### 2.6. Illustration for a Complex 3D Mechanical Environment: Compression of a Trabecular Bone Sample

As an additional illustration of the procedure in a more complex 3D environment, we simulated a compression of 1% on a trabecular bone sample. The trabecular bone sample was issued from the distal extremity of a porcine femur, and its geometry was obtained by micro-Computed Tomography (μ-CT) (resolution of 9.8 μm). We selected a 1 mm^3^ cube within the CT volume and simulated the compression test with FEBio. Bone tissue was considered to be linearly elastic, with a Young’s modulus of 17 GPa and a Poisson’s ratio of 0.3. The two directions that minimize |λ(A)−1| with A as a unitary vector in the plane tangent to the surface were computed at each surface node.

## 3. Results

### 3.1. Simple Example: Sensitivity to Poisson’s Ratio in Uniaxial Tension

As an illustration of the previous equations, let us consider a case of planar uniaxial tension, for which the right Cauchy-Green tensor may be written in terms of the Poisson’s ratio ν and the applied strain e in the direction $e_{1}$:

(3) $$C={\left(1+e\right)}^{2}e_{1}⊗e_{1}+{\left(1-\nu e\right)}^{2}e_{2}⊗e_{2}$$

The calculation of stretch in any direction $A=\cos\theta \;e_{1}+\sin\theta \;e_{2}$ is thus straightforward:

(4) $$\lambda =\sqrt{\cos^{2}\theta {\left(1+e\right)}^{2}+\sin^{2}\theta {\left(1-\nu e\right)}^{2}}$$

where θ stands for the angle between the direction A and the stretching direction $e_{1}$.

**Figure 1:**
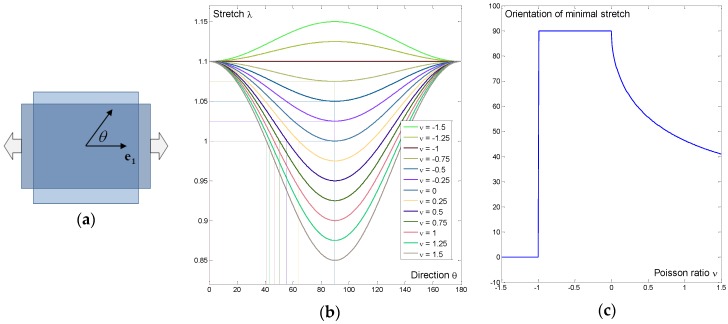
Illustration of the minimal stretch calculation in the case of two-dimensional uniaxial stretch. (**a**) Description of the orientation angle on a membrane subject to uniaxial tension; (**b**) Stretch as a function of the orientation with respect to the stretching direction for different values of Poisson’s ratio; (**c**) Orientation of the minimal stretch or unitary stretch as a function of Poisson’s ratio. Interestingly, a discontinuity is observed for auxetic material with a Poisson’s ratio of −1.

By minimizing the quantity |λ−1|, the angle of minimal stretch is easily obtained and is expressed as a function of the applied strain and Poisson’s ratio. Therefore, the sensitivity of the computed minimal stretch to Poisson’s ratio can be assessed, as illustrated in Figure 1 for the particular case e=0.1 and for Poisson’s ratio in the range [−1.5, 1.5], therefore including auxetic cases. 

Obviously, for auxetic materials, no direction corresponds to unitary stretch since the substrate is elongated in every direction. In this case, we only considered the direction in which the stretch was minimal. Interestingly, we observed that the orientation of minimal stretch is discontinuous for auxetic cases, with a preferred direction going from 90° when Poisson’s ratio is in the range [−1, 0] to 0° when Poisson’s ratio is lower than −1. 

### 3.2. Planar Case: Stretching of a Membrane

**Figure 2:**
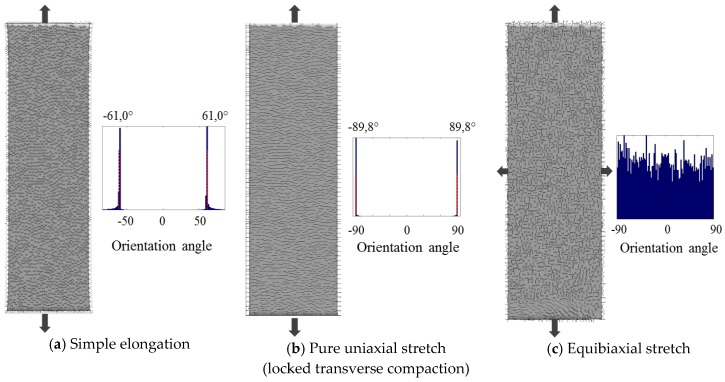
Computed preferential orientation of cells seeded on an elastic membrane under different stretch conditions. (**a**) Simple elongation (free transverse compaction); (**b**) Pure uniaxial stretch (locked transverse compaction); (**c**) Equibiaxial stretch. The results are in accordance with the reported results [11].

In Figure 2, we represented the computed preferential orientation of cells seeded on a membrane subject to a 10% strain. Three cases were distinguished as proposed in [11]: (1) simple extension; (2) pure uniaxial stretch; (3) equibiaxial stretch. Unsurprisingly, there was no preferential orientation in the case of equibiaxial stretch since the stretch is equal in every direction. In the case of pure unitary stretch, the predicted orientation was found to be perpendicular to the stretching direction (orientation angle equals to ±89.78°). The stretch is indeed unitary in this direction due to locked transverse compaction. These two results are in accordance with the reported results [11]. In the case of simple extension, two mirror-image angles were computed with values of ±61.03°, in accordance with numerous reported studies [9,10,11,12,13,14]. 

### 3.3. Physiological Case: Inflation of an Arterial Segment

**Figure 3:**
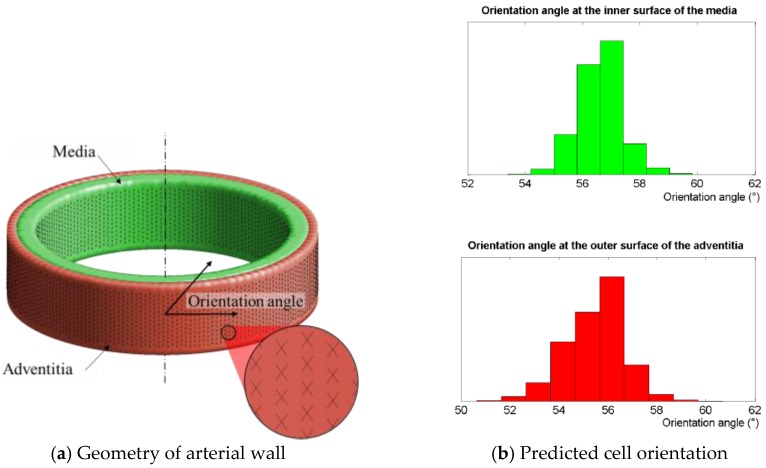
Computed cellular orientation in the physiological case of the inflation of an arterial segment. Orientation angles (defined as the angle between computed preferential orientation and the transverse plane of the arterial segment) were computed at the inner surface of the media and the outer surface of the adventitia. Values were respectively ±56.6° (±0.8°) at the inner surface of the media and ±55.5° (±1.2°) and the outer surface of the adventitia, to be compared with the values around 50° used when modelling the anisotropic structure of the collagenous structure of the artery [23].

The preferential cellular orientations were computed at the inner surface of the media and the outer surface of the adventitia after the simulation of the inflation of an arterial segment (see Figure 3). We found mean angles between the predicted cell orientation and the transverse plane of the arterial wall of ±56.6° (±0.8°) at the inner surface of the media and ±55.5° (±1.2°) and the outer surface of the adventitia, respectively. These computed orientations are to be compared with the experimental values of 51.2° and 40° used for the media and adventitia, respectively, in the modelling of the anisotropic structure of the arterial wall [23]. The underlying assumption in this statement is that the cellular orientation within a loaded structure may be strongly correlated with the anisotropy of the collagen network, as proposed in the literature [6].

### 3.4. Illustration for a Complex 2D Mechanical Environment: Stretching of a Perforated Membrane

**Figure 4:**
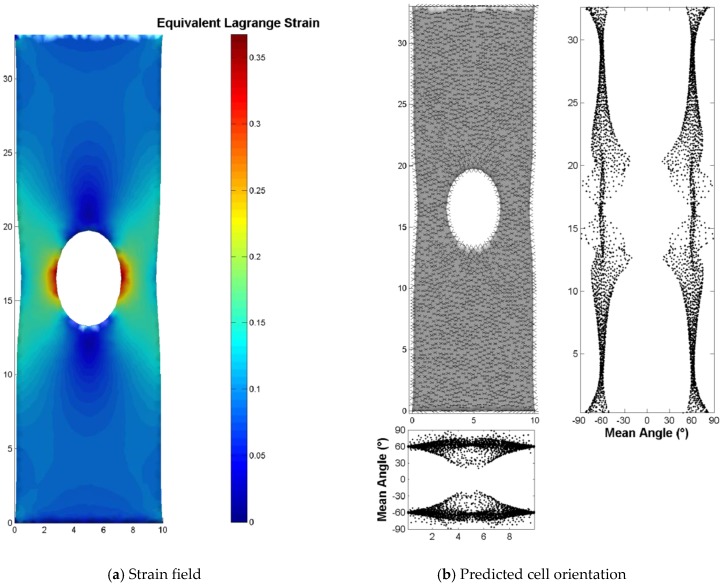
Illustration of the procedure enabling the computation of cell orientation in the case of a complex 2D strain field that is experimentally testable. (**a**) Uniaxial stretch of a perforated membrane leads to a heterogeneous strain field in the neighborhood of the hole; (**b**) Mirror-image angles are heterogeneously distributed on the membrane surface.

As shown in Figure 4, the uniaxial tensile test simulated on a perforated membrane led to heterogeneous strains in the neighborhood of the hole. The equivalent Lagrange strain was represented at the surface of this membrane, as well as the predicted cellular orientation. We observe that this heterogeneous strain field involves heterogeneous cellular orientation. Far from the neighborhood of the hole, the orientation was unsurprisingly similar to the case of simple extension reported above (homogeneous orientation around 60°). 

### 3.5. Illustration for a Complex 3D Mechanical Environment: Compression of a Trabecular Bone Sample

**Figure 5:**
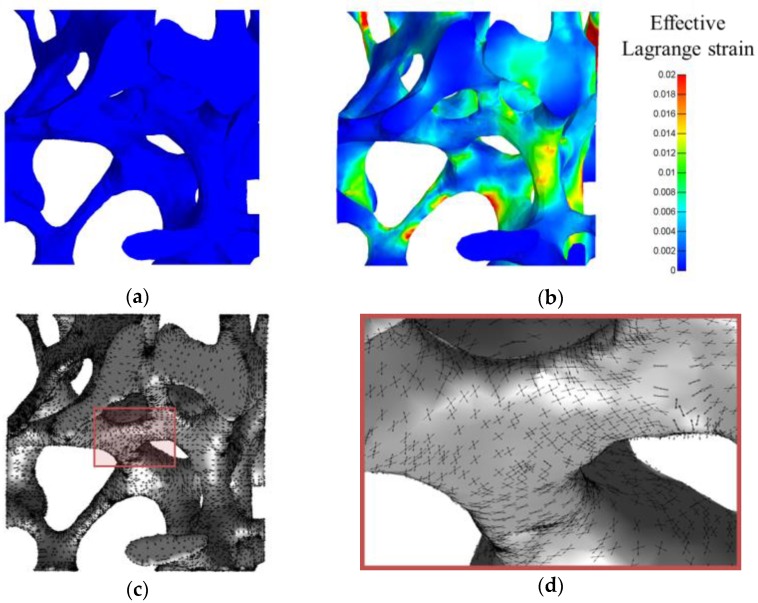
Computed cellular preferential directions computed at the surface of a trabecular bone sample subject to a uniaxial 1% compression test. Color code corresponds to effective Lagrange strain before (**a**) and after (**b**) the compression. Two cellular preferential directions were computed for each mesh node, as visible for the whole sample on (**c**) and for a specific trabeculae on (**d**).

An additional illustration regarding the determination of cell orientation in a complex 3D mechanical environment is given in Figure 5, in which a 1% compression of a trabecular bone sample has been modelled. This compressive force locally corresponds to either tensile or compressive stimuli, and corresponds to the trabecular bone physiological loading. The strain field is obviously largely heterogeneous in this case, and two preferential orientations have been predicted at each node of the mesh. As far as these directions are involved in the creation of an anisotropic collagen network [6], the results of this simulation are a starting point for further simulation of the creation of a collagenous extracellular matrix. It is important to emphasize that we simulated a case that is mostly applied to tissue engineering: cells are seeded at the surface of a solid scaffold, and are dynamically stimulated in order to encourage tissue growth. To extend this simulation to more realistic bone tissue, cells could be initially placed within both the mineral phase and the inorganic extracellular matrix. The cellular preferential direction would then be computed in a similar way, by finding the unitary stretch for any 3D direction. 

## 4. Discussion

The reorientation of cells adhered on a substrate subject to external mechanical loadings has been studied experimentally and mathematically for the last two last decades. However, the proposed models such as the “zero-strain theory” have never been generalized to 3D structures, potentially with a complex constitutive law, and subject to combined mechanical loadings. Instead, these models have only been exploited in the case of simple stretch conditions on linear elastic 2D substrates. In the present paper, we presented and discussed a simple and novel procedure to predict preferential cell orientation in a given mechanical environment, based on the results of common FE analysis. The main advantage of the proposed hypothesis is its simplicity and versatility, as it may be applied to any 3D substrate subject to any complex mechanical load, without requiring the identification of fitting parameters. 

In a first illustration, we observed that cell orientation may exhibit a particular response to auxetic materials when the Poisson’s ratio is around −1. The interest and existence of such auxetic materials have been largely described and studied, including in our group [28,29]. Performing dynamic cell cultures on such materials, that are potentially 3D-printed at the sub-micron scale from a definition of its microstructure, may therefore constitute an interesting topic for studying the cellular response to a particular mechanical environment. The numerical implementation of our simple assumption has then successfully predicted the reported observations concerning cell orientation in the case of simple extension, pure uniaxial stretch, and equibiaxial stretch [14]. It is worth noting that the proposed approach may not be applicable in the case of very soft substrates, for which the tension in the cytoskeleton is not sufficient to maintain focal adhesions. In this case, it has been indeed observed that the cells did not align in the same direction than that for the stiff substrates [30]. The procedure was then applied to the computation of cellular preferential directions within a two-layer arterial wall subject to physiological pressure. Interestingly, the computed directions were satisfyingly aligned with the directions of the collagen fibres in the anatomic structure of an arterial wall, with different values for the media and adventitia consistent with those considered in the relevant literature. It may indicate that these preferential cellular directions have strong implications as far as the anisotropy of the secreted collagen network is concerned, which is consistent in the reported results [6]. 

In the second part, we provided two illustrations of the versatility of the procedure in the case of 2D and 3D complex mechanical environments. The simple case of the uniaxial stretching of a perforated membrane enables us to obtain a heterogeneous strain field, and is easily testable experimentally. Experimental observations in this case may result in rapid validation or rejection of the present model. It will also provide precious information concerning the cell orientation in the case of heterogeneous combined tensile/compressive strains. These experiments are currently carried on with perforated and non-perforated membranes using a dynamic bioreactor designed by our group [31]. Moreover, these computed directions may have implications concerning the direction of collagen fibers during the healing of circular wounds, which has been very recently studied from computational approaches [27]. It has indeed been reported that collagen fibers surrounding a healing wound were clearly aligned in preferential directions [26] that may correspond to the computed directions of minimal stretch presented above. 

Finally, the procedure was applied to compute preferential directions at the surface of 3D geometry corresponding to trabecular bone. This procedure may be particularly applied to the culture of cells within computer-aided designed scaffolds for tissue engineering. Indeed, FE analysis has often been performed on 3D porous scaffolds [32,33] and more recently by our team on ligament fibrous scaffolds [34,35]. From the present procedure, the preferential cell directions at the surface of these scaffolds may be easily computed from the calculation of the strain tensor field issued from FE analysis. The initial cell orientation constitutes crucial information in further simulation of collagen network formation within a scaffold, since cell orientation is directly linked to the direction of the cell-produced collagen matrix [6].

The present hypothesis obviously has limitations, since the cellular direction in a given environment certainly depends on numerous other factors, such as the gradient of concentration of biochemical substances, the cellular phenotype, and most importantly the presence, amount, and type of the neighbouring cells. Indeed, one underlying assumption in the present work is that cells respond independently to the substrate stretch, which requires that cells are plated at low density. It is likely that the reported results cannot be extended to the case of confluent cells, as cell-cell communication should affect the cellular preferential direction [7,10]. In the case of confluent cells, different approaches (stochastic simulations [36], reaction-diffusion equations [37], or other mathematical methods [38]) are used to model cell population dynamics. Moreover, it has been clearly observed that the cellular orientation also depends on the substrate curvature (this phenomenon has been modelled recently [39]), or on the micro- or nano-structured surfaces [40]. These aspects may be added in the future to the assumption formulated here. However, the illustrations proposed in the present work correspond to specific cases where the substrate curvature may be neglected as long as the curvature is largely higher than the size of a cell, and where subcellular-size topography may be neglected. These two assumptions are verified in most of the common studies in which cellular orientations are studied under dynamic stimulation. 

While several studies have studied the dynamics of cell reorientation during the first hours of stretching [8], as well as the effect of strain frequency [7] or waveform [9], it is worth noting that in the present work we only focused on the final orientation of cells when a cyclical load is applied with no consideration of the specificity of the loading cycle. The pathways by which the cells sense the substrate stretch were not addressed within the present contribution. Nevertheless, without entering into the details of mechanotransduction, the assumption made in the current study may simply be interpreted using this reasoning: (1) cells require a level of stretch (sometimes called basal strain energy [14]) in order to adhere onto the substrate; (2) lengthening a cell involves strain energy within its stress fibers, energy which is minimized during homeostasis; (3) shortening a cell causes the required level of stretch to vanish, as long as actin filaments cannot bear compression and thus buckle [14]. 

Despite these obvious limitations, the present work constitutes a first attempt to propose a general model for the computation of cellular preferential directions in various 3D scaffolds that are subject to a complex mechanical load, and constitutes a milestone in the simulation of the creation of an anisotropic collagen network in a given mechanical environment. This point, on which our team actively focuses, may enable us to predict the evolution of mechanical properties due to extracellular matrix formation in the framework of tissue engineering, which is one of the most challenging tasks in the design of a suitable substitute for specific clinical applications. 

## 5. Conclusions

In conclusion, the assumption that cells align in the direction of unitary stretch (computed in any environment from the right-Cauchy Green tensor) was assessed and validated in the present work. A simple method was reported to compute such directions, and the results were compared to the previously reported experimental data. New insights concerning future experiments to be performed were exposed, and some elements indicating that cellular direction may be the basis of the orientation of collagen fiber secretion were detailed. This work constitutes a first step to the formulation of a deeper understanding of the orientation of cells within or at the surface of any 3D scaffold subject to any complex load.
